# Comparison of Profile Similarity Measures for Genetic Interaction Networks

**DOI:** 10.1371/journal.pone.0068664

**Published:** 2013-07-10

**Authors:** Raamesh Deshpande, Benjamin VanderSluis, Chad L. Myers

**Affiliations:** Department of Computer Science and Engineering, University of Minnesota - Twin Cities, Minneapolis, Minnesota, United States of America; University of South Florida, United States of America

## Abstract

Analysis of genetic interaction networks often involves identifying genes with similar profiles, which is typically indicative of a common function. While several profile similarity measures have been applied in this context, they have never been systematically benchmarked. We compared a diverse set of correlation measures, including measures commonly used by the genetic interaction community as well as several other candidate measures, by assessing their utility in extracting functional information from genetic interaction data. We find that the dot product, one of the simplest vector operations, outperforms most other measures over a large range of gene pairs. More generally, linear similarity measures such as the dot product, Pearson correlation or cosine similarity perform better than set overlap measures such as Jaccard coefficient. Similarity measures that involve L_2_-normalization of the profiles tend to perform better for the top-most similar pairs but perform less favorably when a larger set of gene pairs is considered or when the genetic interaction data is thresholded. Such measures are also less robust to the presence of noise and batch effects in the genetic interaction data. Overall, the dot product measure performs consistently among the best measures under a variety of different conditions and genetic interaction datasets.

## Introduction

Similarity measures are among the most important operations used in analyzing genomic data. One of the most widely used analysis paradigms, guilt-by-association, requires measuring similarity between gene pairs or other objects of interest based on a high-dimensional set of features. Guilt-by-association has proven particularly important for the analysis of genetic interactions because similarity of genetic interaction neighbors of two genes is often easier to interpret than direct interactions between genes [1]–[3]. A genetic interaction is a measure of how surprising a double gene knock-out phenotype is, compared to the phenotype expected from known single gene knock-out phenotypes [4]. Using this definition, genetic interactions can be quantitatively measured [5], but often occur between genes that lack an obvious close functional relationship [2]. Because of the difficulty in understanding and interpreting individual genetic interactions, they are frequently studied in the context of other interactions in the dataset. For example, the complete list of a gene’s interactions, commonly referred to as a profile, can be compared with other profiles, and similarities between the gene profiles are indicative of functional similarity [1], [5].

Understanding profile similarity measures for genetic interactions and other related datasets has become increasingly critical in the recent years because several large-scale or whole-genome genetic interaction studies have been published in several organisms including baker’s yeast (*Saccharomyces cerevisiae*) [1], [6], fission yeast (*Schizosaccharomyces pombe*) [7]–[9], bacteria (*Escherichia Coli)* [10], fly (*Drosophila melanogaster*) [11], worm (*Caenorhabditis elegans*) [12] and human (*Homo sapiens*) [13]. Many of these high-throughput studies use profile correlation for validation and interpretation of the generated genetic interaction data, or discovery of new gene functions. Despite its importance, no systematic study has been conducted to compare the efficacies of different profile similarity measures on genetic interaction data using biological relevance as a benchmark. Nevertheless, there are studies in other fields where correlation measures have been compared either from a theoretical standpoint [14], [15] or for specific applications such as co-citation data analysis [16], [17], co-localization detection in visualization [18], chemical fingerprint searches [19], microarray gene expression profile analysis [20], and cluster analysis [21], [22]. Most of these applications either involve datasets that are binary in nature, such as the co-citation study, or involve datasets that consist of only positive continuous elements such as pixel scores. In contrast, genetic interaction data are continuous and signed. Other data that are continuous and signed, such as gene expression data, are fundamentally different in that genetic interaction networks tend to contain much sparser signal and the different signs in genetic interaction data have very different biological interpretations [1], [5]. The unique properties of genetic interaction data warrant a separate and systematic study of the correlation measures.

In this study, we systematically benchmarked several similarity measures on two of the largest genetic interaction datasets [1], [9]. Further, we investigated how measurement noise and batch effects, which commonly affect genetic interaction assays [5], impact the performance of these similarity measures. We also address the effect of thresholding continuous genetic interaction data, a common practice in this field [1], [2], [5], [23], on the performance of the different similarity measures.

## Results and Discussion

We compared a diverse set of profile similarity measures that cover a range of classes including linear similarity measures, set-overlap measures, rank based similarity measures, hybrid measures, and L_2_ normalization based measures (see Table 1). We also included a similarity score specifically developed for genetic interactions (COmplex or linear Pathway, COP score; ) [6], [24] and a recently developed similarity score that works well for discovering several non-linear relationships (Maximal Information Coefficient, MIC) [25]. For a baseline comparison, we use the product of degrees of the two profiles [26].

**Table 1:** Similarity measures evaluated in this study.

	Correlation	Formula (x, y)	Description	Study
1	Pearson	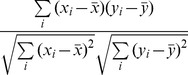	Linear similarity measure that uses mean-centering and normalization of the profiles.	Pearson 1920 [29]
2	Cosine	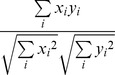	Linear similarity measure that uses normalization of the profiles.	
3	Spearman	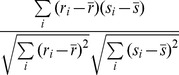 where 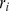 is rank of 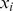 in x, 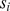 is rank of 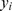 in y.	Spearman correlation is Pearson correlation on the ranks of elements in the profile.	Spearman 1904 [34]
4	Overlap negative	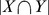 Where 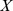 and 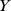 are set of significant negative interactors for x and y respectively.	A set overlap measure	Similar to Russell and Rao’ measure 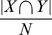 where N is size of the profile [35].
5	Overlap positive	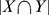 Where 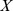 and 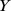 are set of significant positive interactors for x and y respectively.	A set overlap measure	Similar to Russell and Rao’ measure 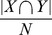 where N is size of the profile [35].
6	Overlap p-value	-log_10_(Hypergeometric p-value of the overlap of X and Y), where 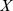 and 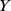 are set of significant interactors for x and y respectively.	A statistically relevant set overlap measure	
7	Jaccard	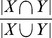	A set overlap measure	Jaccard 1901 [36]
8	Gini	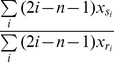 where 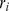 is rank of 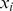 in x, 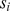 is rank of 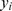 in y.	A hybrid measure between Pearson and Spearman correlations.	Schechtman [30]
9	Dot Product	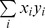	A linear similarity measure.	
10	Maximal Information Coefficient (MIC)	See citation.	A coefficient designed to discover a wide range of relationships including non-linear relationships.	Reshef 2011 [25]
11	COmplex or linear Pathway (COP) score	See citation.	A similarity measure developed specifically for genetic interactions.	Collins 2006, 2007 [6], [24]
12	Baseline degree product	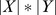 Where 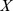 and 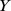 are set of significant interactors for x and y respectively.	A baseline similarity measure based on the degrees of the two genes in the network.	

We used two genetic interaction datasets from two different species and laboratories: Costanzo *et al*. from *S. cerevisiae* [1] and Ryan *et al.* [6] from *S. pombe* to evaluate these similarity measures.

Our primary basis for evaluation of the similarity measures was their ability to discover known functional relationships between genes. More specifically, each metric was used to rank a set of gene pairs based on their interaction profile similarities, and the highest-scoring gene pairs were compared with a functional co-annotation standard developed using the Gene Ontology (GO standard; see Methods: Creating GO standard) [27], [28]. The similarity measures were evaluated using precision-recall analysis, which is a continuous way of evaluating top similarities at different similarity cutoffs with the GO standard. Precision refers to the percentage of similarities at a given threshold that correctly associate functionally related gene pairs, while recall refers to the proportion of all functionally related gene pairs among the set of top similarities. Similar strategies have been used previously in evaluating other kinds of genomic data or prediction methods (e.g. see [28]).

### Comparison of Different Correlation Measures on Genetic Interaction Data

**Figure 1:**
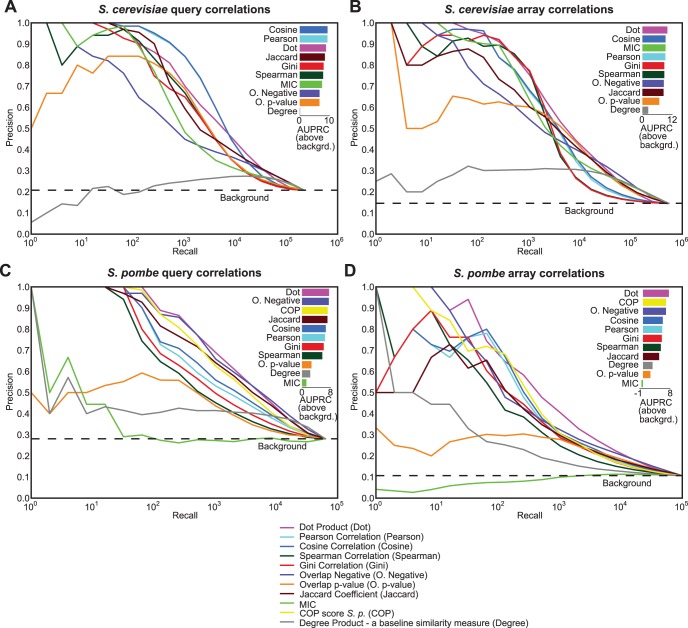
Comparison of similarity measures applied to genetic interaction datasets. Gene pair correlations derived from each similarity measure were benchmarked against a Gene Ontology-based standard using precision-recall statistics. The comparison was conducted on (A) *S. cerevisiae* genetic interaction data (Costanzo *et al.* 2010) - query genes’ similarities, (B) *S. cerevisiae* genetic interaction data - array genes’ similarities, (C) *S. pombe* genetic interaction data (Ryan *et al.* 2012) - query genes’ similarities, and (D) *S. pombe* genetic interaction data – array genes’ similarities. The horizontal dotted line shows the background precision expected from randomized ranking of gene pairs. The bar plot on the upper right corner in each section shows the area under the precision-recall curve (AUPRC) above the background for each similarity measure. The area was calculated by summation of the areas of trapezoids at increments of 2^n^ (log_2_ units). The bars are sorted by their respective areas above background.

Overall, the dot product was the most consistent top performer in terms of its precision-recall characteristics on both the *S. pombe* and *S. cerevisiae* genetic interaction datasets for both query (rows) and array (columns) genes’ correlations. Pearson correlation [29], which is the most widely used profile similarity measure for genetic interactions, performed well for low recall (Figure 1). Cosine correlation, also known as un-centered Pearson correlation, shows precision-recall performance close to that of Pearson correlation (Figure 1). This similarity in performance is expected as the means of genetic interaction profiles are very close to zero, which makes the two metrics equivalent. High precision at low recall appears to be a general property of similarity measures that normalize profiles, such as Pearson, cosine, Spearman and Gini correlations. Another aspect common to these correlations is that their performance drops sharply for higher recall (Figure 1). In contrast, metrics that do not normalize genes’ profiles, such as overlap-negative and the dot product, do not experience a similar dramatic drop in performances at high recall. Gini correlation [30], which is considered a hybrid between Pearson and Spearman correlation, performed similarly to or slightly better than Spearman but its precision was worse than Pearson correlation’s precision at any choice of recall (Figure 1).

MIC, a general correlation measure theoretically capable of discovering a wide variety of relationships, performed non-randomly but worse than most other similarity measures on the *S. cerevisiae* data. On the *S. pombe* data, the MIC performed slightly worse than the background (random) expectation, which is surprising. This low performance may be because MIC might be emphasizing technical artifacts in the data (e.g. batch effects [5]) not related to the biological signal. Another notable disadvantage of MIC is that it is computationally very expensive to compute all pairwise correlations on genetic interaction matrices using this measure. For example, the MIC tool takes almost one month on one processor (2.3 GHz, 16 GB RAM) to compute all pairwise correlations on the Costanzo *et al.* 2010 genetic interaction matrix, compared to approximately 1 second to compute the dot product on the same data. Therefore, it was not possible to consider MIC in subsequent analyses.

The COP score is a correlation measure developed specifically for clustering and understanding genetic interaction data. The original COP score was based on a combination of log-logistic probabilities of Pearson correlation of the profiles and direct interaction between the genes. The parameters for the log-logistic function were determined from a gold standard of protein complexes [6], [24]. One limitation of the original COP score is that it requires interactions between all pairs of genes, so it can be applied only to symmetric genetic interaction matrices, such as the one published in Collins *et al.* 2007 [6]. In the Ryan *et al. S.pombe* study [9], the authors addressed this limitation by using only the Pearson correlation component of the COP score. We used the *S. pombe* similarity matrix published in Ryan *et al.*, 2012 [9], which was calculated using the modified COP score, for the evaluations here. We find that the modified COP score performs better than the Pearson correlation but is slightly worse than the dot product (Figure 1C, D).

There is a possibility that the top profile similarities are driven solely by the degrees of the gene profiles [26], trivially favoring high similarities between pairs of hub genes. To address this concern, we included the product of the negative genetic interaction degrees of two genes as a baseline similarity measure between hubs. Our evaluations show that this measure is slightly better than background but is much worse than most similarity measures for both *S. cerevisiae* and *S. pombe* data (Figure 1). This result confirms that the similarity measures are discovering relationships between genes that cannot be trivially predicted using genetic interaction degree alone.

**Figure 2:**
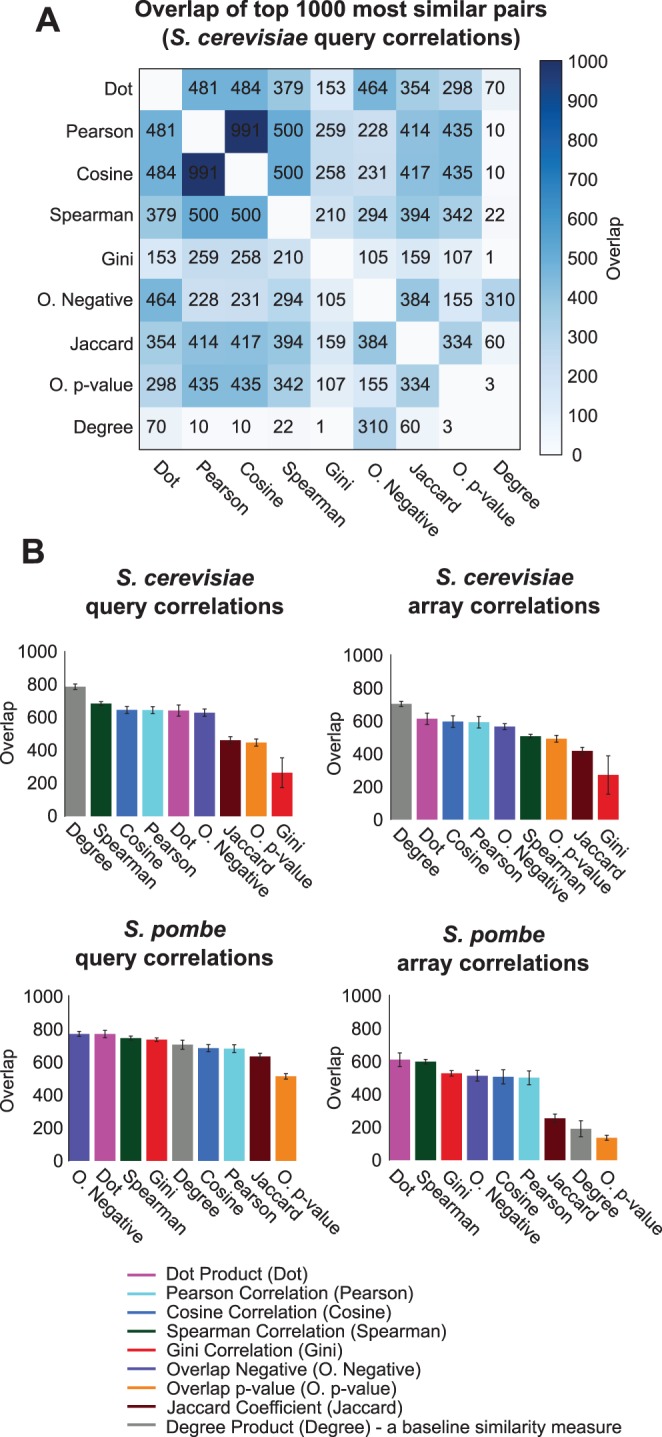
Comparison of similarity measures based on highly similar gene pairs and stability on partial data. (A) The overlap of the top 1000 similar gene pairs for each similarity measure were compared against each other on the query gene side of the *S. cerevisiae* genetic interaction data (B) The stability of the similarity measures was assessed by comparing the overlap of top 1000 similar gene pairs computed using 10 different random selections of 50% of the data in each profile.

Are profile similarity measures discovering the same or different top most similar gene-pairs? To answer this question, we compared the ranked lists of gene pairs obtained from different similarity measures. We find that the top gene-pairs are similar across similarity measures yet they are not completely identical. For example, the maximum overlap between top 1000 most similar gene-pairs for any two similarity measures (except Pearson-cosine) is at most 500 (Figure 2A). This observation is surprising considering that several similarity measures exhibit similar precision-recall performance (Figure 1). This observation suggests that all similarity measures are not identical even though their precision-recall performances may be and there is considerable diversity between the various measures.

Stability of a similarity measure when partial profiles are used is important as often genetic interactions profiles are incomplete. For example, for the *S. cerevisiae* genetic interaction screens, the query side is incomplete as screens are still ongoing, and for the *S. pombe* screens, both query and array sides are incomplete. Gene pairs assessed as similar by the similarity measures on partial profiles would ideally not dramatically change as these profiles are completed. To quantitatively assess the stability of the similarity measures, we computed gene similarities using partial profiles generated by randomly selecting 50% of the genetic interaction data. For example, 50% of the array genes were randomly selected to compute similarities between query genes. This random selection and computation of similarities was conducted 10 times, and the top 1000 similar gene pairs were compared across different selections. We observe that linear similarity measures, Dot product, Cosine and Pearson are the most stable similarity measures (Figure 2B). Spearman correlation and overlap negative are similar in performance. Other similarity measures are either not consistent across all datasets or are consistent only for a few cases. For example, Overlap p-value and Jaccard coefficient are the least stable similarity measures across different datasets. Gini correlation is an example of a similarity measure that is consistent only for *S. pombe* genetic interactions but is the least stable similarity measure for *S. cerevisiae* dataset.

### Thresholding Effects

**Figure 3:**
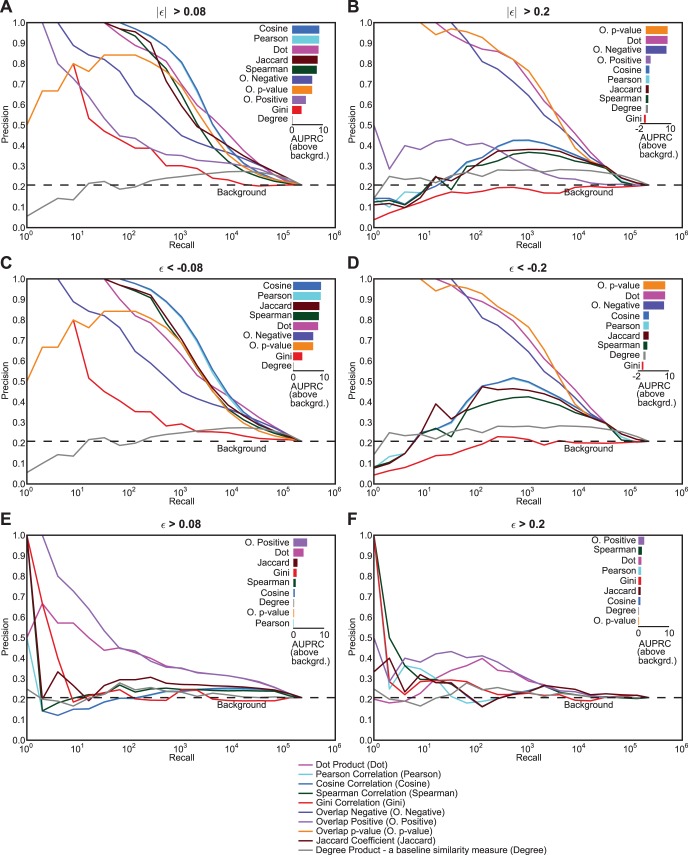
Role of thresholding genetic interaction data in the performance of similarity measures. The precision-recall plots were compared on the query side of the *S. cerevisiae* genetic interaction data at several thresholds (A) ε<−0.08 - only negative genetic interactions at intermediate threshold, (B) ε<−0.2 - only negative genetic interactions at a stringent threshold, (C) ε >0.08 - only positive genetic interactions at an intermediate threshold, (D) ε >0.2 - only positive genetic interactions at a stringent threshold, (E) |ε| >0.08, negative and positive interaction at an intermediate threshold, and (F) |ε| >0.2, negative and positive interaction at a stringent threshold. The bar plot on the upper right corner in each section shows the area under the precision-recall curve (AUPRC) above the background for each similarity measure. The area was calculated by summation of the areas of trapezoids at in increments of 2^n^ (log_2_ units). The bars are sorted by their respective areas above background.

Thresholding of the genetic interaction data by considering only interactions greater than a certain score and setting the rest to zero is a common way of removing weaker and noisier interactions [1], [5]. Although thresholding focuses the analysis on the stronger signal, it may have undesirable effects depending on which profile similarity measure is used. To check the effect of thresholding on similarity measures, we systematically evaluated the similarity measures for several cases of thresholding on the query genes’ correlation of Costanzo *et al.* data (Figure 3; refer to Figure 1A for no thresholding case).

When absolute thresholding was used (intermediate: |ε| >0.08; stringent: |ε| >0.2; Figure 3A,B) we find that the precision-recall performance of the similarity measures that normalize gene profiles (Pearson, cosine, Spearman and Gini correlations) decreases dramatically after stringent thresholding (Figure 3 A,B). On the other hand, non-normalizing correlation measures (for example, dot product and overlap-negative) were robust to thresholding (Figure 3). In fact, overlap-negative and overlap p-value’s performances improve with the stringency of the threshold cutoff. When only negative interactions were considered (intermediate: ε<−0.08; stringent: ε<−0.2; Figure 3 C,D), the performance was more or less similar to absolute thresholding for most similarity measures. However, when only positive interactions were considered (intermediate: ε >0.08; stringent: ε >0.2), we found that only the overlap and the dot product showed performances better than random expectation (Figure 3E,F). This suggests that negative genetic interactions are the main driver for most correlation measures.

**Figure 4:**
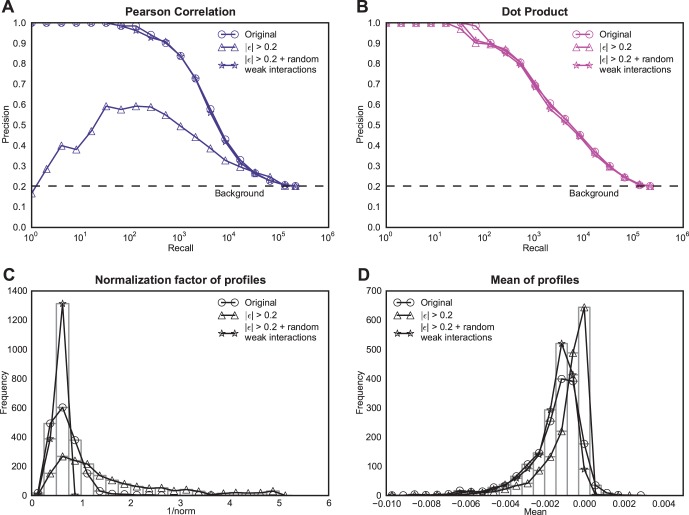
Investigation of Pearson correlation relative to the dot product for thresholded genetic interaction data. In each of the panels, three instances of genetic interaction data have been used: original data, original data with all interactions whose absolute value was less that 0.2 set to zero, and original data where all interactions whose interaction value is less than 0.2 reorganized randomly. The three data instances are investigated using (A) the precision-recall performance of Pearson correlation on each instance, (B) the performance of dot product on the same three instances, (C) a histogram of normalization factor (1/norm) of the profiles for the three instances, and (D) a histogram of the mean of profiles for the three instances.

The drop in the performance of some similarity measures on thresholded data raises the question of whether there is any functional signal in the weak interactions, and whether that signal is contributing to better performance of these measures when not thresholded. To address this question, we reassigned the weak interactions (|ε| <0.2) by randomly shuffling their values among themselves. After randomizing, we were surprised to observe that the performance of Pearson correlation increased to the near original performance (Figure 4A). In contrast, the dot product is neither affected by the thresholding nor by the random shuffling of the weak interactions (Figure 4B) suggesting that high dot product similarity is not driven by weak interactions. Both of these observations clearly suggest that the weak interactions have little or no biological information in them.

To find out what caused the sensitivity of the Pearson correlation to thresholding, we investigated two differences between Pearson correlation and dot product: mean centering of the profiles and L_2_ normalization of the profiles. Pearson correlation mean centers the profiles, which may affect the strength of all interactions and disrupt the performance. However, we rule out this possibility because we observed that the mean of a genetic interaction profile is normally very close to zero (Mean of means across gene profiles = 0.002 =  = 1% of the 0.2 threshold used; Figure 4D). Furthermore, cosine correlation, which does not mean-center the profiles, exhibits similar precision-recall performance to Pearson correlation (Figure 1, 2). Therefore, the second difference, L_2_ normalization of the profiles, is the main factor in Pearson correlation’s sensitivity to thresholding. When Pearson correlation was used on the thresholded data, the normalization factor in the Pearson correlation (1/L_2_ norm of the profile) greatly varied compared to the normalization factor in the non-thresholded data (Figure 4C). This variance in the normalization factor means that the interactions in the hub profiles, which have larger norms, are multiplied by smaller normalization factors, and on the other hand, non-hubs, because of smaller norms, are multiplied by a larger normalization factor. This difference in the normalization leads to a relative magnification of the interaction values in non-hub profiles, making them falsely similar to many other profiles. When the interactions are not thresholded or when random noise is added to the data, the weak interactions or noise serve as filler that equalizes the normalization factor across all profiles (Figure 4C). This equalization of the normalization factor effectively protects the Pearson correlation from low degree genes exhibiting spurious high correlations. This equalization of the normalization factor also makes it behave much like the dot product, which assumes a uniform normalization factor (normalization factor = 1; see [14] for a discussion on linear measures and their normalization factors).

### Simulated Noise

To assess the robustness of the similarity measures to noise in the genetic interaction measurements, we added three kinds of noise to the *S. cerevisiae* data: false negatives, false positives, and additive Gaussian noise. For false negatives, we assigned 95% of the interactions in the data to zero which is equivalent to removing interactions from the data; for false positives, we sampled real interactions and placed 10 times their number in place of non-interactions; and for additive noise, we added randomly sampled Gaussian noise with 0 mean and 0.08 standard deviation to the *S. cerevisiae* data (0.08 is the intermediate interaction cutoff for Costanzo *et al.* data). Query genes’ correlations from the *S. cerevisiae* datasets were evaluated after simulated noise was added.

**Figure 5:**
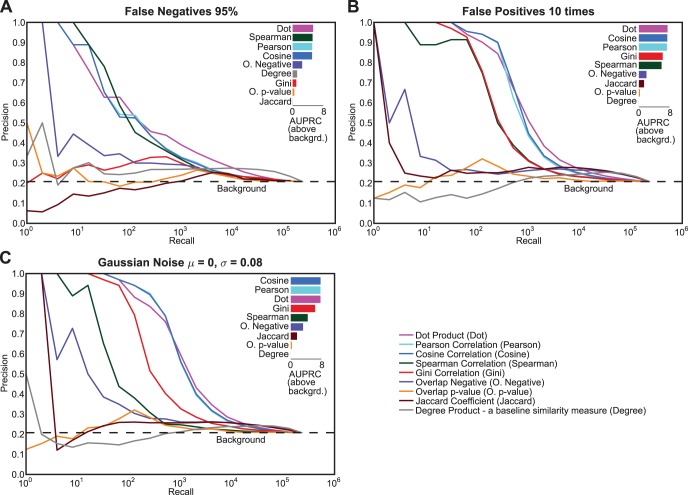
Role of noise in the genetic interaction data on similarity measure performance. In each panel, simulated noise was added to the *S. cerevisiae* genetic interaction data, and query correlations were used for comparing the similarity measures. The simulated noise conditions are (A) false negatives –95% of the significant interactions whose absolute value of interaction is greater than 0.08 were randomly set to 0, (B) false positives – values were randomly sampled from the set of genetic interactions whose absolute interaction value were greater than 0.08 and were randomly substituted in place of randomly selected non-interactions. This random sampling was repeated until 10 times the number of significant interactions were added as false positives in the original data, and (C) Gaussian noise - random values from a Gaussian distribution of mean 0 and standard deviation 0.08 were added to all values (interactions and non-interactions) in the dataset. The bar plot on the upper right corner in each section shows the area under the precision-recall curve (AUPRC) above the background for each similarity measure. The area was calculated by summation of the areas of trapezoids at in increments of 2^n^ (log_2_ units). The bars are sorted by their respective areas above background.

As expected, all types of noise adversely affect the precision-recall performance of all similarity measures (Figure 5). However, the dot product measure appears to be the most robust similarity measure to all three noise conditions (Figure 5). Surprisingly, overlap-negative, which is a binary version of the dot product on negative genetic interactions, performs poorly in the noise conditions. The two other linear similarity measures, Pearson and cosine correlation, perform well at low recall but the precision-recall performance drastically decreases at higher recall (Figure 5). In contrast, Jaccard coefficient and overlap p-value are the most affected in all three noise conditions; their performances drop dramatically from near best performance on the original dataset to almost random performance in the noise conditions. Gini correlation is also strongly influenced by noise, but seems to be most sensitive to false negatives as it performs reasonably well for the false positives and Gaussian noise conditions.

### Batch Effects

High-throughput genetic interactions are adversely affected by batch effects, traces of which may still remain in spite of normalization methods for removing them [5]. To determine how batch effects influence the profile similarity measures, we simulated batch effects in the *S. cerevisiae* genetic interaction data by randomly creating batches of 5 query genes and to each profile in the batch we added a common bias for each gene sampled from a Gaussian distribution with mean, μ = 0 and standard deviation, σ = 0.02 (μ = 0, σ = 1 for Ryan *et al.* 2012), and further, we added Guassian noise (μ = 0, σ = 0.02 or 1) to the entire dataset. We simulated more serious batch effects by doubling the magnitude of both the batch signal and the standard deviation of the Gaussian noise added to the entire dataset. Query correlations were analyzed using precision-recall performances to understand the impact of batch effects.

**Figure 6:**
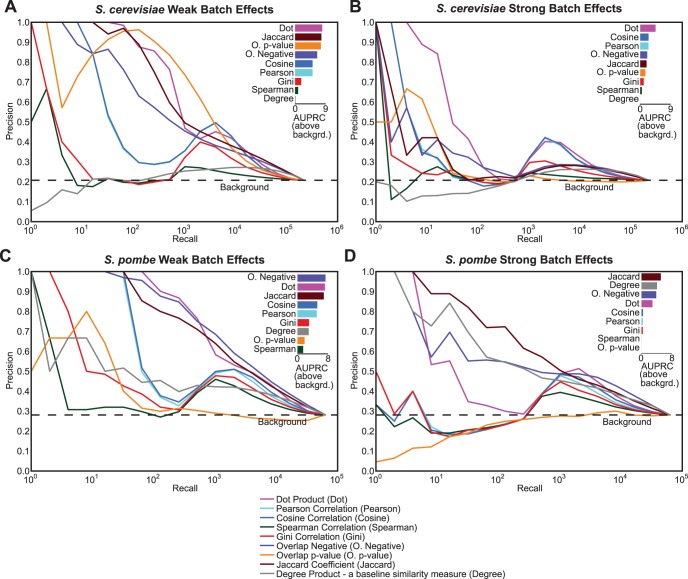
Role of simulated batch effects in genetic interaction data on similarity measure performance. (A) shows the performance of similarity measures on the query side of the *S. cerevisiae* genetic interaction network when simulated intermediate batch effects were added to the data. The batch effects were added by creating random batches of size 5 and for each batch, Gaussian noise (μ = 0 and σ = 0.02) was added. Furthermore, Gaussian noise (μ = 0 and σ = 0.02) was added to entire dataset. (B) A stronger batch effect signature and noise was added (μ = 0, σ = 0.04 for both batch effect and noise) (C), (D) are similar plots for the query side of the *S.pombe* genetic interaction data (μ = 0, σ = 1 for (C), and μ = 0, σ = 2 for (D)). The bar plot on the upper right corner in each section shows the area under the precision-recall curve (AUPRC) above the background for each similarity measure. The area was calculated by summation of the areas of trapezoids at in increments of 2^n^ (log_2_ units). The bars are sorted by their respective areas above background.

We find that batch effect conditions are destructive for most similarity measures, especially those that include normalization (Figure 6). This reduction in performance is because normalization magnifies the batch signature for profiles with fewer interactions, and therefore, falsely predicts genes with weak profiles in the same batch to be functionally similar (Figure 6).

Other similarity measures that do not use normalization perform much better. For example, we observe that the dot product and Jaccard similarity measures are robust to batch effects. The robustness of Jaccard coefficient to batch effects is surprising given that it was one of the most sensitive similarity measures to different noise conditions. The overlap p-value is another surprising candidate: it performs poorly in the noise conditions (Figure 5) but is the best performer for query correlations of Costanzo *et al.* data under intermediate batch effect conditions (Figure 6A). However, the performance of overlap p-value is not consistent for stronger batch effects or for the *S. pombe* dataset (Figure 6B,C,D). When the batch effect was severely increased, all similarity measures break down in terms of their performance (Figure 6B,D).

There is a peak in the precision-recall performance for higher recall for Pearson, cosine and dot product which corresponds to the point at which all within-batch gene-pairs are exhausted (Number of within batch gene-pairs for 1800 query genes in *S. cerevisiae* 1800/5 * 5 choose 2 = 3600).

## Conclusion

Profile comparison is a widely used approach for genetic interaction studies. Profile similarity measures are used to validate genetic interaction data [5], construct functional profile similarity maps [1], and predict gene functions or drug targets [1], [31]. We routinely use Pearson or cosine correlations to cluster genetic interaction data for viewing in clustergram format, and we often look at several versions of the genetic interactions data some of which are thresholded. In this study, we have shown that some similarity measures may not reflect reliable gene-associations when applied to thresholded data, so we need to be aware of this vulnerability. Furthermore, similarity measures have varying sensitivities to different noise and batch effects conditions, so controlling for these conditions may require the use of specific similarity measures.

The dot product is one of the most consistent performers across all datasets and conditions evaluated here. Furthermore, it seems to be more robust to most noise conditions and batch-effects compared to other similarity measures. The dot product can be seen as a hybrid between cosine correlation and overlap measures: applying dot product on binarized data results in the overlap measure and applying dot product on normalized (L_2_ norm) data produces the same result as cosine correlation. Indeed, the dot product seems to combine the best properties of these two measures. For instance, cosine correlation performs well on the full dataset but its performance degrades as the threshold on the data is increased. The overlap measure, on the other hand, improves with an increase in the stringency of the threshold and performs well at higher recalls. The dot product is able to retain the good performance of cosine correlation at low recall, but is not affected by thresholds applied to the data and also retains good performance at higher recall. Furthermore, the dot product is among the fastest profile operations computationally, so it is also attractive from that perspective. However, there are a few disadvantages of dot product, the most significant being that unlike several of the other correlation measures, its value is not directly interpretable as it is not bounded (e.g. between −1 and 1). Thus, choosing the right threshold for analysis is highly dataset specific and depends on the size and scale of the values in the interaction dataset. Another disadvantage is that dot product may not be readily available in commonly used clustering softwares. We have tried to address this disadvantage by implementing a dot product measure in cluster 3.0 [32], which is frequently used in combination with Java Treeview [33] for cluster visualization. Our implementation is freely available and can be downloaded from http://csbio.cs.umn.edu/people/RaameshDeshpande/profileSim.

For future work, we propose to develop approaches for combining top similarities from different methods. This proposal is based on the observation that different correlation measures detect different top similarities that all seem to provide relatively high performance in predicting functional associations. This suggests the potential for developing a combined measure that is able to combine the strengths of the different measures to provide superior performance.

## Materials and Methods

### Creation of GO Standard

The *S. cerevisiae* Gene Ontology (GO) standard was created using the approach described in [28], which generates a co-annotation matrix based on the *S. cerevisiae* Gene Ontology [27] and annotations. Both of the datasets were downloaded on January 22, 2012. The final standard includes annotations for all pairs of 5513 genes with some denoted as positives (functionally related), some as negatives (not functionally related), and some as zero (neither). The GO standard for S. *pombe* was created in a very similar manner to *S. cerevisiae* and contains 4598 genes. The *S. pombe* Gene Ontology data was downloaded on July 4, 2012. The GO standards for both species are available for download on the Supplementary website (http://csbio.cs.umn.edu/people/RaameshDeshpande/profileSim).

### Precision-Recall Analysis

Pairwise similarities were calculated for all genes appearing in each genetic interaction dataset, except for pairs between multiple alleles of the same gene (multiple alleles for some genes were screened in Costanzo *et al.*), in which case the allele with highest degree was chosen. The calculated gene similarities were sorted and precision and recall were calculated at different similarity thresholds, above which similarities were considered to be positive predictions. These predictions were compared with gold standard positive and negative sets derived from Gene Ontology [28]. For programmatic analyses, an all genes by all genes GO standard matrix is created where values in the matrix are 1, 0 or −1 depending on whether the two genes are co-annotated, undetermined and definitely not co-annotated in Gene Ontology respectively. A prediction that matches with 1 in the GO standard is a True Positive, −1 is a False Positive, and 0 s are undetermined and therefore ignored. Pairs with a 1 in the GO standard, but a similarity score below the threshold are considered False Negatives. Precision is given by TP/(TP+FP) and recall by TP/(TP+FN), where TP, FP, FN stand for numbers of True Positives, False Positives and False Negatives respectively. In this study, we have used Recall = TP because the denominator TP+FN is equal to number of 1 s in the GO standard, which is a constant.

### Genetic Interaction Datasets

We used two genetic interaction datasets, one from *S. cerevisiae* and another from *S. pombe*. Similarities between the replicates or different point mutation alleles of the genes with other genes could confound precision-recall performance analysis. So in both the datasets, in case of replicates or multiple alleles of the same gene, we have retained the profile with the highest negative interaction degree (ε<−0.08, pval <0.05). The details of each dataset after processing are as follows:

1. *S. cerevisiae:* Costanzo *et al.* Synthetic Genetic Array (SGA) genetic interaction dataset (after removing replicates) dimensions are 1672 query (rows) by 3885 array (columns) genes.
2. *S. pombe*: Ryan *et al.* Epistatic MiniArray Profiles (EMAP) genetic interaction dataset (after removing replicates), dimensions are 879 query (rows) genes by 1955 array (columns) genes.
